# The impact of vocabulary assessments on quality of life: Insights from professionals on their application with students with disabilities

**DOI:** 10.1371/journal.pone.0313690

**Published:** 2024-11-12

**Authors:** Faisl Alqraini, Khalid Alasim, Abdulaziz Alqahtani

**Affiliations:** 1 Department of Special Education, Prince Sattam Bin Abdulaziz University, Al-Kharj, Saudi Arabia; 2 Department of Special Education, Taif University, Taif, Saudi Arabia; Faculty of Electrical Engineering, Computer Science and Information Technology Osijek, CROATIA

## Abstract

Assessing vocabulary skills is a crucial aspect of educational interventions for students with disabilities, as it directly influences their academic progress, overall communication abilities, and quality of life. This study aims to explore professionals’ perceptions regarding the vocabulary assessments used with students with disabilities. By gaining insights into their perspectives and experiences, we can improve the assessment process and enhance instructional practices, ultimately contributing to a better Quality of Life (QoL) for these students. Data were collected through a survey questionnaire completed by 375 professionals working in the field of special education. The findings indicate that professionals in the private sector express higher satisfaction levels and demonstrate better proficiency in applying vocabulary assessment tools compared to those in the public sector. Furthermore, the results reveal that professionals specializing in speech and language disorders report higher satisfaction levels compared to professionals in other specialized areas.

## Introduction

Students with disabilities often confront substantial challenges in language acquisition and vocabulary development. These difficulties can arise from various factors, including cognitive impairments, language disorders, and learning disabilities [1]. Consequently, it is imperative to assess and monitor their vocabulary growth to identify areas needing additional support and intervention. Vocabulary acquisition is fundamental for reading comprehension, as understanding 90% to 95% of the vocabulary within a text is essential to avoid reading impairments and comprehension deficits due to insufficient vocabulary proficiency [2]. A limited vocabulary during early childhood is linked to significant risks for negative social and academic outcomes [3]. Effectively addressing these challenges not only enhances academic performance but also substantially improves the overall Quality of Life (QoL) for students with disabilities by fostering better communication and social integration [4].

Research indicates that a student’s annual vocabulary acquisition should range from 2,000 to 3,000 words [5]. During the elementary stage, students should acquire approximately 25,000 words. Those with vocabulary proficiency below this threshold are considered linguistically delayed. Standardized tests and measures are employed to assess vocabulary development at each educational stage [5]. Furthermore, vocabulary development significantly impacts QoL. An extensive vocabulary enhances communication skills, academic performance, and cognitive abilities, thereby facilitating better social interactions and broader opportunities in life.

Professionals working with students with disabilities, including special education teachers, speech and language pathologists, and educational psychologists, play a pivotal role in selecting appropriate vocabulary assessments. These professionals possess specialized knowledge and expertise relevant to the unique needs and capabilities of students with disabilities [6]. They meticulously consider various factors, such as individual student needs, the nature of their disabilities, and the assessment’s objectives, to ensure that the chosen assessment tools are optimally aligned with this population’s specific requirements.

Research suggests that traditional vocabulary assessments may not accurately capture the vocabulary skills of students with disabilities [7]. These assessments often rely heavily on written responses or timed tasks, which may not accommodate the diverse abilities and learning styles of students with disabilities. As a result, professionals may employ a range of alternative assessment methods, such as visual supports, manipulatives, or technology-assisted assessments, to better capture the vocabulary skills of these students [7].

Understanding the views of professionals on the appropriateness and effectiveness of these measures is key to realizing educational goals and QoL for students. In this light, this study was designed to determine satisfaction levels of professionals regarding the appropriateness of the vocabulary assessment measures. It was set to find out if these measures were sufficient in supporting their professional roles and whether they helped them achieve their educational goals, thus contributing to an improved QoL for the students they serve. By studying their perceptions, one would be able to understand more about the weaknesses and strengths of the current measures for vocabulary assessments and the modifications that need to be made to ensure better educational achievements and quality of life for students with special needs.

This investigation aims to address two primary research questions:

1. What is the degree of satisfaction among professionals regarding vocabulary assessments for students with disabilities?
2. Which independent variables—specifically, professional specialization, work environment, education level, and years of professional experience—demonstrate statistical significance in elucidating the variance in professionals’ satisfaction levels concerning vocabulary assessments?

### Vocabulary assessment

There are established norm-referenced tools for assessing receptive and expressive vocabulary [8]. Two commonly used standardized tests are the Peabody Picture Vocabulary Test (PPVT) [9] and the Expressive Vocabulary Test (EVT) [10]. The PPVT evaluates receptive vocabulary through picture identification, allowing an examination of comprehension skills without the need for expressive demands [9]. In contrast, the EVT assesses expressive vocabulary by having students name pictures or provide word definitions orally [10].

### Sample vocabulary assessments used with students with disabilities

#### Peabody picture vocabulary test

The Peabody Picture Vocabulary Test (PPVT) [9] is the most commonly utilized instrument for assessing receptive vocabulary. The PPVT is an individually administered, norm-referenced measure of receptive vocabulary for hearing individuals aged 2 years and 6 months through adulthood. During PPVT administration, examinees are required to identify, from four image options, the picture that corresponds to a spoken target word provided by the examiner. For deaf and hard-of-hearing students who utilize simultaneous communication (i.e., spoken English paired with Manually Coded English Systems), target words are presented through both oral and manual modalities. Extensive data support the validity of the PPVT for assessing hearing students; however, the normative sample contains very limited representation of deaf and hard-of-hearing individuals [11].

#### British picture vocabulary scale

The British Picture Vocabulary Scale (BPVS) is one of the popular tests that assess the receptive vocabulary of children and adolescents. This scale assesses the ability to understand spoken English words as individual pictures. Each test item consists of a word spoken by the examiner and four pictures from which the participant then selects the picture that best describes the word. The BPVS has been utilized by psychologists, speech and language therapists, and researchers for various purposes, such as identifying language development levels, diagnosing language impairment, and tracking development. It can also be applied in both educational and clinical contexts or even research contexts; hence, it is a robust tool that enables its users to understand and assist in language development [12].

#### The Hodson assessment of phonological patterns

The Hodson Assessment of Phonological Patterns, Third Edition (HAPP-3), is a diagnostic tool utilized by speech-language pathologists to evaluate and diagnose phonological patterns associated with individuals, including children, afflicted with a speech sound disorder. Created by Barbara W. Hodson, HAPP-3 helps determine the severity and types of phonological deviations that guide the preparation of effective intervention programs [13]. HAPP-3 is a well-rounded test that allows the clinician to assess a wide variety of phonological processes, including but not limited to consonant deletion, cluster reduction, stopping, fronting, and gliding [14]. It has been validated for reliability and stability in identifying phonological disorders and encompasses a phonological sorting form with numerous words to ensure a detailed analysis [14].

Another striking feature of HAPP-3 is the use of specific vocabulary items in the assessment procedure. The phonological sorting form is a word list that acts as a stimulus to elicit and sample phonological patterns and the resulting deviations within spontaneous speech. This vocabulary-driven approach ensures that many varied phonological patterns are assessed. This, in turn, helps the clinician make judgments related to appropriate target patterns that HAPP-3 can remediate. For example, in a case study of a highly unintelligible child, specific phonological patterns were targeted for remediation cycles to hasten intelligibility gains [13].

#### Expressive one-word picture vocabulary test

The Expressive One-Word Picture Vocabulary Test (EOWPVT) was developed to evaluate expressive vocabulary in hearing students by labeling pictured objects, actions, and concepts. The EOWPVT-4 is an individually administered, norm-referenced instrument appropriate for examinees aged 2 years to over 80 years [11]. According to the authors, nearly two decades of research support the concurrent and predictive validity of the EOWPVT-4 for assessing hearing children.

#### Carolina picture vocabulary test

The Carolina Picture Vocabulary Test (CPVT) [15] is the only vocabulary assessment with normative data for deaf and hard-of-hearing students [11]. The CPVT is a 130-item instrument that measures receptive sign vocabulary in deaf and hard-of-hearing students who primarily communicate using manual signing. Norms were developed based on 767 individuals aged 2 years 6 months to 16 years. The normative sample consisted of those with prelingual deafness, a better-ear hearing threshold of 80 dB or greater, an IQ between 80 and 100, normal-hearing parents, and primary reliance on manual signing for communication. However, the 2 years 6 months to 4 years age band included only 18 children, and the 5 years age band contained just 19 children. Therefore, Layton and Holmes advised cautious interpretation of CPVT results for young children, given the limited sample size [16]. During administration, examinees must identify from four options the picture representing the word signed by the examiner. Standardization utilized solely sign language presentation by examiners. The general format of the CPVT closely parallels that of the Peabody Picture Vocabulary Test, an oral receptive vocabulary measure for hearing students [16].

#### Grammatical analysis of elicited language-presentence level

The Grammatical Analysis of Elicited Language-Presentence Level (GAEL-P) was developed at the Central Institute for the Deaf in St. Louis, Missouri. The instrument consists of three components: readiness skills, single words, and word combinations [17]. The single-word section assesses deaf and hard-of-hearing students’ ability to comprehend and articulate 30 common, early-acquired object nouns (e.g., shoe, ball, boat) [18]. To evaluate expressive vocabulary, the examiner individually presents each of the 30 objects and prompts the student to verbally label each one [11]. The GAEL-P was normed on both typically hearing children and children with hearing loss. The hearing sample consisted of 200 children between 2 years and 6 months and 5 years of age. The hearing loss sample comprised children between 5 and 9 years old who utilized an oral educational approach [17].

Professionals in special education and speech-language pathology aim to ensure that vocabulary assessments for students with disabilities are accurate and informative for instructional planning. These assessments, when properly selected and administered, can significantly impact students’ quality of life by informing targeted interventions. Professionals consider students’ linguistic backgrounds, cognitive abilities, and communication needs when choosing assessments. By analyzing professionals’ experiences and recommendations, we can refine assessment practices and develop more effective strategies for vocabulary development. This, in turn, can lead to improved educational outcomes and enhanced quality of life for students with disabilities, affecting their social interactions, academic achievements, and future opportunities.

### What this paper adds?

This paper offers a comprehensive examination of vocabulary assessment practices for students with disabilities, focusing on the perspectives of professionals in the field. It uniquely bridges the gap between assessment tools, professional satisfaction, and quality-of-life outcomes for students. By comparing satisfaction levels across different sectors and specializations, the study provides novel insights into the effectiveness of current assessment methods. The research highlights the limitations of traditional assessments and emphasizes the need for more inclusive approaches. Its findings have significant implications for professional development, assessment tool refinement, and educational policy. Ultimately, this study contributes valuable empirical evidence to inform improvements in vocabulary assessment strategies, aiming to enhance both educational outcomes and overall quality of life for students with disabilities.

## Materials and methods

### Population and participants

Participants were chosen using random sampling. The sample included Saudi citizens employed in both government and private sectors, working with disability populations across the country. Specifically, these participants were recruited from schools, hospitals, and rehabilitation centers through their departments. There was no direct communication between the participants and the researchers, only through communication with the departments’ heads. To determine the right sample size, G*power statistical software analysis was used. This software had a medium effect size, a power of 0.80, an alpha of 0.05, and a 0.05 error probability to improve the statistical validity of the potential, using the Cohen [19] standard. An adequate sample size of at least 180 special education specialists was necessary to detect an effect in the difference between specialists attributed to their demographic characteristics (majority, workplace, level of education, and years of experience), if that effect exists.

As Table 1 shows, three hundred seventy-five professionals who work with students with disabilities throughout Saudi Arabia completed an electronic questionnaire that was made available via the Google Form website. One hundred and ninety-two (51.2%) of the respondents were females, while one hundred and eighty-three (48.8%) were males. The majority of respondents (43.7%, n = 164) were in the private sector, 31% (n = 119) were in the government sector, and 24.5% (n = 92) were in the hospital sector. This may occur because the participants from private sectors recruit via social media accounts for their centers as their main points of contact beside the electronic email address.

**Table 1:** Demographic characteristics of participants.

Characteristic	Number and Percentage
**Workplace (*n* = 375)**	
**Government sector**	119 (31.7%)
**Private sector**	164 (43.7%)
**Hospital**	92 (24.5%)
**Educational level (*n* = 375)**	
**Graduate level (MA & PhD)**	59 (15.7%%)
**Bachelor**	208 (55.5%)
**Diploma**	108 (28.8%)
**Years of experience (*n* = 375)**	
**Less than 1 year**	45 (12.0%)
**1–5 years**	138 (36.8%)
**5–10 years**	133 (35.5%)
**More than 10 years**	59 (15.7%)
**Major**	
**Speech and Communication Disorders**	185 (49.3%)
**General Special Education**	164 (43.7%)
**Deaf and Hard of Hearing**	26 (6.93%)

Participants’ educational levels were as follows: 55.5% (n = 208) had a bachelor’s degree, 28% (n = 108) had some college (diploma after earning a BA), and 15.7% (n = 59) had a graduate degree (MA or Ph.D. or both). With regard to major, half of the participants (49.3%, n = 185) reported speech and communication disorders. Additionally, 164 (43.7%) reported general special education, and 26 (6.9%) reported being deaf or hard of hearing. The professionals in these three majors expect to use more language assessments, such as vocabulary, due to their nature of work assessing and evaluating individuals’ language and literacy, while most of these individuals have faced challenges in language since birth, such as DHH [20–23]. Most of the respondents (84.3%, n = 316) had less than 10 years of experience working with disabled students. It may seem, based on the current data, that employment in the last ten years has a higher rate compared to the past, while the majority of professionals who responded to this questionnaire have less than ten years of experience.

### Measures and procedures

Quantitative data were gathered through a questionnaire from professionals in Saudi Arabia who work with disability populations. This questionnaire was called the Satisfaction Questionnaire of Vocabulary Assessment (SQVA). All the items were created by the authors based on a review of the literature on vocabulary assessment and special education (e.g.,) [8, 24–31].

The Satisfaction Questionnaire of Vocabulary Assessment (SQVA) begins by informing participants about the study’s purpose, the confidentiality of their information, and how to answer the questions. The second section includes questions about participants’ demographic information, such as their major, workplace, level of education, and years of experience. The third section comprises nine items that were adapted from relevant studies [8, 27–31]. Participants rated their challenges with vocabulary assessment services on a 5-point Likert scale ranging from “Very Dissatisfied” to “Very Satisfied.” The Arabic version of the questionnaire takes approximately 15 minutes to complete.

### Validity

Content validity. To ensure the external validity of the instrument, the accuracy of the SQVA questionnaire was evaluated before distribution. It was reviewed by a group of four experts in the field of disability in Saudi Arabia, who provided feedback on the nine items in the questionnaire. The authors requested their feedback on the clarity of the SQVA instrument, the relevance of each item to its linguistic and cultural appropriateness, and the extent to which all items addressed the significance of vocabulary assessment services. The authors made minimal changes, including rewording certain items for enhanced clarity while maintaining the overall instrument as the focal point to avoid unnecessary length. The authors developed an electronic questionnaire after reviewing the instrument and considering expert feedback and suggestions. This process helped ensure the SQVA instrument’s appropriateness for capturing vocabulary assessment practices and services within the Saudi sector context.

### Construct validity

The SQVA scale was subjected to exploratory factor analysis (EFA). Prior to conducting EFA, the suitability of the data for factor analysis was evaluated. The correction matrix was examined, and many coefficients with values of .3 and above were detected. EFA using the principal analysis (PF) method was conducted. The minimum factor loading criteria were set to 0.50. The Bartlett’s Test of sphericity proved to be significant, and all communalities were over the required value of 0.500, which indicates its suitability for factor analysis. Consequently, the number of items in the final version of the questionnaire is 9, which confirms the validity of the questionnaire, allowing it to be used in the current study as indicated in Table 2.

**Table 2:** Correlation coefficients of the questionnaire items.

No	Items	*r*	Sig.
**1**	The official vocabulary assessments are easy to use.	.391^a^	.001
**2**	The vocabulary assessments measure the actual level of the student.	.402^a^	.001
**3**	Clarity of instructions on how to use the vocabulary assessments.	.458^a^	.001
**4**	The vocabulary assessments facilitate parents’ recognition of their children’s language needs in acquiring vocabulary.	.336^a^	.001
**5**	The vocabulary assessments are electronically automated.	.394^a^	.001
**6**	The vocabulary assessments contribute to identifying the strengths and weaknesses of the students in language.	.372^a^	.001
**7**	The results of the vocabulary assessments are effectively employed in building the appropriate educational and therapeutic program for the students.	.366^a^	.001
**8**	Currently, the translated and standardized vocabulary assessments are outdated.	.315^a^	.001
**9**	The vocabulary assessments are suitable for the Saudi environment.	.426^a^	.001

^a^Correlation coefficient function at the 0.05 level for two-tailed.

### Reliability

Sample size plays a crucial role in determining the reliability of Cronbach’s alpha coefficients. Research suggests that reliability estimates become stronger with larger sample sizes [32, 33]. The reliability literature often suggests that a minimum sample size of 200–300 is required for coefficient alpha [34–36]. In this study, based on an alpha of 0.05, the minimum sample size requirement is 180 to detect at least 80.0% of the power of the test. However, the total sample size of 375 participants was obtained in this study. Therefore, the reliability of the scale was determined through Cronbach’s alpha, where there were sufficient subjects to measure it. The 9-item SQVA showed sufficient reliability for all scores in this study, r = .94. The mean inter-item correlation is .70, with values ranging from .58 to .79. This suggests quite a strong relationship among the items [37, 38]. Thus, the SQVA is a reliable measurement instrument for this study.

### Data collection

After the Institutional Review Board approval was obtained, the electronic survey was distributed to the participants following the outlined procedures. An official letter was dispatched by the scientific research deanship of the authors’ affiliated university to the Ministry of Education, requesting the distribution of consent forms, along with the electronic questionnaire, to the General Administration of Education nationwide. The target sample comprised special education teachers in schools and professionals working with special needs individuals in the private sector, including deaf and hard of hearing (DHH), learning disability (LD), speech disorders (SD), intellectual disability (ID), and autism. We dispatched follow-up letters, emails, and WhatsApp messages to officials in the General Administration of Education in every region to ensure the intended sample received the electronic questionnaire. The authors provided details about the participants’ voluntary participation, safeguards to protect their information, data handling procedures, and the researchers’ contact details.

A second procedure was used to recruit more participants via social media, particularly from the private sector, because most of them have social media as one of their main ways to make contact. The authors conducted a thorough search of all private sectors that offer services for special needs students, using their electronic addresses. We sent WhatsApp messages to the directors of private sectors across the KSA, inviting them to share the questionnaire link with their qualified professionals for study participation. This letter described the purpose and importance of the study to potential participants, the duration of data collection, and the availability of the link.

The recruitment period for this study began on February 2, 2024, and ended on March 23, 2024. After this three-week period, the authors collected all of the completed surveys because no more participants were received and a larger sample size than required by G*power software (at least 180 participants) was obtained.

### Data analysis

The section followed several steps. First, to prevent duplicate responses or to confirm the authenticity of the respondents, duplicate data were identified and eliminated through the use of unique identifiers, such as participant IDs, to ensure that each study participant was represented once in the dataset [39]. To ensure the accuracy and consistency of the data, over 20% of the incomplete survey items were classified as partial data and removed [40].

Second, descriptive statistics on all demographic data were computed. Then the frequency, percentage, and rating averages for the participants’ level of satisfaction with the vocabulary assessments using a 5-point Likert scale (1 = very dissatisfied to 5 = very satisfied) were calculated. Based on the calculation used to distribute professionals’ satisfaction levels on a five-point scale (1–5 = 4, 4/3 = 1.33), professionals’ satisfaction levels were divided into three categories: (a) high satisfaction ranging from 3.67 to 5.00, (b) average satisfaction ranging from 2.34 to 3.66, and (c) low satisfaction ranging from 1.00 to 2.33 [41]. Likert scale cutoff determination was used to rate professionals’ satisfaction levels, which included high professionals’ satisfaction (from M = 3.67 to 5.00), moderate professionals’ satisfaction (from M = 2.34 to 3.66), and low professionals’ satisfaction (from M = 2.33 and below).

Third, before analyzing the data to answer question two, the assumptions for conducting the ANOVA test were reviewed. The statistical assumptions of the parametric ANOVA test were assessed, and all assumptions were met for three independent variables (majors, workplace, and years of experience). Only one independent variable did not meet the assumptions of the parametric ANOVA test (educational levels). Consequently, the analysis was conducted using a one-way ANOVA and a non-parametric Kruskal-Wallis test. The one-way ANOVA was used to determine if the professionals’ level of satisfaction regarding the vocabulary assessments differed based on their majors, workplace, and years of experience. However, the Kruskal-Wallis H test was used to determine if the professionals’ level of satisfaction for the vocabulary assessments differed based on their level of education. Finally, the Statistical Package for the Social Sciences program (SPSS version 22.0) [38] analyzed all the data.

## Results

Research question 1. *What is the degree of satisfaction among professionals regarding vocabulary assessments for students with disabilities*?

The results show that the overall mean score for teachers’ and professionals’ satisfaction with all vocabulary assessment items was 2.43 (SD = .36; range 2.08–3.65), reflecting an average degree of satisfaction. Furthermore, the mean score for professionals’ satisfaction with the usage and implementation of vocabulary assessment was 2.14 (SD = .66; range 2.08–2.21), reflecting a low degree of satisfaction. The mean score for professionals’ satisfaction with the accuracy and usefulness of the vocabulary assessment service for determining the actual level of all students’ vocabulary was 2.25 (SD = .71; range 2.24–2.26), reflecting a low degree of satisfaction. However, the mean score for professionals’ satisfaction with the instructions and accessibility of vocabulary assessment was 2.41 (SD = .73; range 2.34–2.49), reflecting an average degree of satisfaction. The mean score for professionals’ satisfaction with measuring and employing vocabulary assessments for elementary school students was 2.36 (SD = .75; range 2.34–2.39), reflecting an average degree of satisfaction. Although the mean score for professionals’ satisfaction with the standardized vocabulary assessment was 3.65 (SD = .99), reflecting an average degree of satisfaction, they felt it was suitable for the Saudi environment and culture with a low degree of satisfaction (M = 2.33; SD = .98) (see Table 3).

**Table 3:** Professionals’ satisfaction with vocabulary assessments by means: Highest to lowest.

N	Survey Item	Mean	SD	Satisfaction Level
**1**	The official vocabulary assessments are easy to use.	2.08	.86	Low
**2**	The vocabulary assessments measure the actual level of the student.	2.25	.96	Low
**3**	Clarity of instructions on how to use the vocabulary assessments.	2.34	.102	Average
**4**	The vocabulary assessments facilitate parents’ recognition of their children’s language needs in acquiring vocabulary.	2.21	.99	Low
**5**	The vocabulary assessments are electronically automated.	2.48	1.05	Average
**6**	The vocabulary assessments contribute to identifying the strengths and weaknesses of the students in language.	2.26	1.03	Low
**7**	The results of the vocabulary assessments are effectively employed in building the appropriate educational and therapeutic program for the students.	2.34	1.01	Average
**8**	Currently, the translated and standardized vocabulary assessments are outdated.	3.35	.99	Average
**9**	The vocabulary assessments are suitable for the Saudi environment.	2.33	.98	Average
	Overall, satisfaction of vocabulary assessments.	2.43	.36	Average

Note: Likert ratings were given values ranging from 1 to 5, corresponding to “not very satisfied” to “very satisfied,” respectively. Means are based on these values.

Research question 2. *Which independent variables—specifically*, *professional specialization*, *work environment*, *educational attainment*, *and years of professional experience—demonstrate statistical significance in elucidating the variance in professionals’ satisfaction levels regarding vocabulary assessments*?

First of all, prior to analyzing the data to answer question two, the assumptions for conducting the ANOVA test were reviewed. The statistical assumptions of the parametric ANOVA test were assessed, and all assumptions were met for three independent variables (majors, workplace, and years of experience). Only one independent variable did not meet the assumptions of the parametric ANOVA test (level of education). The assumptions identified in the study design included a continuous dependent variable (professionals’ satisfaction) and four independent variables (majors, workplace, years of experience, and educational levels).

The study satisfied the assumption of independence of observations, as these variables did not exhibit any relationship or participation with each other. The data were assessed for outliers by calculating the Mahalanobis distance to identify multivariate outliers. Outliers are likely to be present in measured continuous variables absent any data entry errors [42]. The Mahalanobis distance results showed that there were no outliers, with p > .001 for each value in the dataset (p = .613 to .979). The assumption of normality was assessed to determine if the dependent variable was approximately normally distributed for each group of variables by conducting the Shapiro-Wilk tests of normality (p > .05).

For the major, workplace, and years of experience variables, the analysis showed that the distribution of professionals’ satisfaction was normally distributed. The significance value was .061, which was more than .05, meaning that the assumption was met. However, the analysis revealed that the distribution of professionals’ satisfaction was not normally distributed for the level of education, with a significance value of .01, which was less than .05, indicating that the assumption was not met.

Second, a one-way ANOVA was conducted to determine if the professionals’ level of satisfaction regarding the vocabulary assessments was different based on their majors. Participants were classified into three groups: professionals who major in deaf and hard of hearing (DHH) (n = 12), professionals who major in speech and communication disorders (SCD) (n = 186), professionals who major in general special education (SPED) (n = 166), and others (n = 8). There were no outliers, as assessed by the boxplot; data were normally distributed for each group, as assessed by the Shapiro-Wilk test (p = .05); and there was homogeneity of variances, as assessed by Levene’s test of homogeneity of variances (p = .81).

As Table 4 shows, a one-way ANOVA analysis found that professionals’ level of satisfaction with vocabulary assessments used for children who are disabled was statistically significantly different among these three groups of majors, F (3,36) = 3.008, p = 0.03. Professionals’ satisfaction score increased from those majoring in speech and communication disorders (M = 2.41, SD = .314), to those majoring in general special education (M = 2.44, SD = .406), and those majoring in deaf and hard of hearing (M = 2.708, SD = .521), in that order.

**Table 4:** One-Way ANOVA for teachers’ and professionals’ level of satisfaction by majors.

Level of Satisfaction	Sum of Squares	df	Mean Square	F	Sig.
**Between groups**	1.198	2	.399	3.008	.030
**Within groups**	48.850	368	.133		
**Total**	50.048	371			

Also, Tukey post hoc analysis revealed that there were only statistically significant differences in the professionals’ level of satisfaction by some majors. For example, a post hoc Tukey HSD test indicated that the mean showed that professionals majoring in deaf and hard of hearing had a statistically significant lower level of satisfaction regarding vocabulary assessment than professionals majoring in speech and communication disorders (Mean Diff = .288; CI = 0.0089–0.569; p = 0.04). However, there was no statistically significant difference between the professionals majoring in deaf and hard of hearing and those majoring in general special education (p = 0.077) or between the professionals in general special education and those majoring in speech and communication disorders (p = 0.905). This might mean that teachers and professionals who work with deaf and hard of hearing do not have enough knowledge and skills in using vocabulary assessment compared to other professionals who majored in speech and communication disorders.

Additionally, a one-way ANOVA was conducted to determine if the professionals’ level of satisfaction regarding the vocabulary assessments differed based on their workplace. Participants were classified into three groups: professionals who work in the government sector (n = 119), professionals who work in the private sector (n = 164), and professionals who work in hospitals with children who have a disability (n = 92). There were no outliers, as assessed by boxplot; the data were normally distributed for each group, as assessed by the Shapiro-Wilk test (p > .05); and there was homogeneity of variances, as assessed by Levene’s test of homogeneity of variances (p = .231). Data are presented as mean ± standard deviation.

The results in Table 5 show that professionals’ level of satisfaction with vocabulary assessments using children who are disabled was not statistically significantly different among workplaces, F (2,37) = 1.77, p = 0.17. Professionals’ satisfaction scores increased from the hospital (M = 2.38, SD = .31), to the private sector (M = 2.42, SD = .37), and the government sector (M = 2.48, SD = .39), in that order (Table 3).

**Table 5:** One-Way ANOVA for professionals’ level of satisfaction by workplace.

Satisfaction Level	Sum of Squares	df	Mean Square	F	Sig.
**Between Groups**	.479	2	.240	1.777	.171
**Within Groups**	50.162	372	.135		
**Total**	50.641	374			

As the assumption of normality was not met for the level of education variable, a non-parametric statistical analysis was conducted using the Kruskal-Wallis H test. A Kruskal-Wallis H test was conducted to determine if the professionals’ level of satisfaction with the vocabulary assessments was different based on their level of education. The educational levels for the participants were classified into three groups: bachelor’s degree (n = 208), diploma degree (n = 108), and graduate degree (n = 59). Table 6: x squared (2) = 0.193, p = 0.908, with a mean rank satisfaction score of 194.63 for a graduate degree, 187.42 for a bachelor’s degree, and 186.70 for a diploma degree. One possible explanation could be that the focus was primarily on theoretical understanding rather than practical application when using vocabulary assessments, or that professionals received the same content and skills regarding the assessments. Therefore, evaluating the curriculum of courses and training programs for professionals in vocabulary assessments could be beneficial in determining if they meet their needs and requirements in their respective roles.

**Table 6:** Kruskal-Wallis H test for professionals’ level of satisfaction by level of education.

Level of Education	N	Mean Rank	χ^2^	*α*
**Bachelor**	208	187.42	.193	.908
**Diploma**	108	186.70		
**Graduate (MA, Ph.D.)**	59	193.63		

In addition, a one-way ANOVA was conducted to determine if the professionals’ level of satisfaction regarding the vocabulary assessments differed based on their years of experience. Participants were classified into four groups of years of experience: less than a year, between 1 and 5 years, between 5 and 10 years, and more than 10 years. There were no outliers, as assessed by boxplot; data were normally distributed for each group, as assessed by the Shapiro-Wilk test (p = .05); and there was homogeneity of variances, as assessed by Levene’s test of homogeneity of variances (p = .301). Data are presented as mean ± standard deviation.

As Table 7 shows, the satisfaction levels regarding vocabulary assessment used by professionals who work with individuals with a disability were not statistically significantly different across years of experience, F (3,371) = .382, p = .766. Professionals’ satisfaction scores increased from the experience of between 5 and 10 years (M = 2.41, SD = .35), between 1 and 5 years (M = 2.43, SD = .34), more than 10 years (M = 2.45, SD = .42), and less than one year (M = 2.47, SD = .38), in that order. A plausible explanation is that access to essential courses and adequate training in using vocabulary assessments remains limited for many professionals. This prevents many professionals from reaping the potential benefits of incorporating vocabulary assessment into their work with special needs students.

**Table 7:** One-Way ANOVA for professionals’ level of satisfaction by years of experience.

Level of Satisfaction	Sum of Squares	df	Mean Square	F	Sig.
**Between Groups**	.156	3	.052	.382	.766
**Within Groups**	50.485	371	.136		
**Total**	50.641	374			

## Discussion

### Overview of results

This study on the satisfaction of professionals regarding the vocabulary assessment for students with disabilities in Saudi Arabia provides a detailed overview of the demographic characteristics and participants’ profiles. This information is important as it helps to understand the professionals involved in the study and sets the stage for interpreting the results that follow. The study includes 375 participants from various sectors, providing a comprehensive range of perspectives within the interdisciplinary field of special education. The presence of significant gender parity guarantees an equitable distribution of representation, thereby promoting inclusivity and mitigating gender-based biases in the research outcomes. The inclusion of a significant number of participants from the private sector in this research underscores the substantial contributions made by private entities in the provision of special education services in Saudi Arabia.

It is of the utmost importance to conduct an in-depth analysis of satisfaction levels within the private sector to gather valuable insights that can inform the development of effective policies and interventions [43]. The prevalence of individuals specializing in speech and communication disorders is consistent with the study’s emphasis on evaluating vocabulary. This allows for a comprehensive examination of the intricacies of language development.

The presence of varying levels of experience among participants, particularly a notable number of early-career professionals who have had limited exposure to vocabulary assessment training, introduced a dynamic factor. These early-career professionals have a unique ability to offer new and innovative viewpoints. However, their limited exposure to training indicates that they may have certain deficiencies in their readiness for vocabulary assessment practices. This is consistent with what was mentioned in the study by Yoon et al. [44], which suggests that when teachers receive significant professional development, it can have a positive impact on students’ achievement. Additionally, Didion et al. [45] conducted a meta-analysis to examine the impact of teachers’ professional development. Didion et al. suggest that when teachers engage in professional development activities focused on improving their instructional practices, it can lead to improved reading outcomes for students.

The research was enriched by the inclusion of these demographic characteristics, as they collectively established a contextual foundation. The presence of diversity in the study ensured that the findings accurately reflected the broader landscape of special education in Saudi Arabia.

The promotion of gender balance contributed to inclusivity, while sector representation recognized the diverse contexts in which special education professionals work [43, p.68-76]. The focus on majors related to speech and communication disorders aligned with the specific research goals. The emphasis on early-career professionals suggested potential opportunities for focused interventions and training initiatives. The participants’ profile section served the purpose of not only providing a description of the participants but also establishing a foundation for understanding the complex dynamics that impact professionals’ satisfaction with vocabulary assessments.

This study raised inquiries regarding the potential influence of sectoral disparities, gender factors, and training exposure on individuals’ perceptions and behaviors. These inquiries served as a foundation for the subsequent examination and understanding of the study’s main findings.

## Research questions: Satisfaction and demographic differences

This study examined the satisfaction levels of professionals in Saudi Arabia regarding vocabulary assessments and analyzed variations based on demographic factors, providing a comprehensive understanding of their perceptions and key findings.

### Satisfaction of professionals

The overall satisfaction of professionals working with vocabulary assessments in Saudi Arabia was found to be moderate. However, distinct patterns emerged, offering valuable insights into key variables affecting satisfaction. A significant finding was the notable lack of satisfaction with the implementation of vocabulary assessments, indicating challenges in using these tools in real-world situations [46]. This highlights the need for further investigation into factors contributing to these challenges and suggests improvements in assessment methods.

Conversely, satisfaction levels for the instruction and accessibility of vocabulary assessment services were moderate, indicating that while professionals face difficulties, they generally find the guidance and accessibility satisfactory [47]. This underscores the necessity for targeted interventions or improvements in specific areas to enhance overall satisfaction with vocabulary assessment practices.

The study revealed that standardized vocabulary assessments might not be fully compatible with the Saudi environment and culture. This finding prompts further exploration into how cultural and contextual factors influence the perceived appropriateness and efficacy of standardized assessments [47]. These insights are crucial for refining assessment tools to better align with local educational needs.

Item-specific analysis showed significant differences in satisfaction levels. Satisfaction with the clarity of instructions and accessibility was average, while lower satisfaction was noted for the ease of use of official vocabulary assessments and the measurement of language proficiency among elementary school students [48]. These disparities highlight specific challenges that professionals face, emphasizing the need for detailed investigation and improvements in these areas.

Overall, this analysis not only evaluates general satisfaction levels but also identifies specific factors impacting professionals’ perceptions. Key factors include the implementation and usage of assessments, clarity of instructions, accessibility, and the suitability of standardized assessments [48]. These findings provide a foundation for targeted interventions, policy adjustments, and training programs to address challenges and improve satisfaction among special education professionals in Saudi Arabia.

### Differences in satisfaction based on demographics

The study also investigated variations in satisfaction levels among special education professionals, considering different demographic factors.

A major finding was the statistically significant difference in satisfaction levels based on professionals’ majors. Those specializing in speech and communication disorders reported higher satisfaction levels, while those in the deaf and hard-of-hearing domains exhibited the lowest satisfaction [49]. This variation suggests that the academic discipline itself may influence how professionals perceive vocabulary assessments. Consequently, customized training programs tailored to address the unique requirements and challenges of each discipline are necessary [49].

The study found no statistically significant differences in satisfaction levels based on the workplace, indicating consistent satisfaction across government, private, and hospital sectors. This uniformity suggests that professionals face similar challenges or successes, regardless of their workplace context [50]. Therefore, interventions to enhance satisfaction can be universally applied without specific contextual modifications.

Additionally, no notable variations in satisfaction levels were found based on educational attainment or years of professional experience, indicating a common viewpoint among professionals regardless of their academic credentials or length of service. This finding underscores the importance of inclusive training programs designed to meet the needs of professionals at different career stages [50]. A consistent approach to training and professional development is crucial for fostering shared expertise and satisfaction in vocabulary assessment practices.

### Impact of training on satisfaction levels among various majors

The lower satisfaction levels observed among professionals majoring in deaf and hard of hearing highlight the potential influence of targeted training [51]. Addressing the specific challenges encountered by each major through customized training content can enhance skill acquisition and foster greater satisfaction. The study found that 80% of participants lacked sufficient training in vocabulary assessment, raising concerns about their preparedness to effectively use assessment tools [52].

Investing in training programs can lead to increased satisfaction levels among professionals, enabling them to navigate their roles more effectively and contribute to positive student outcomes. Policymakers and educational institutions must prioritize initiatives that bridge this training gap and enhance the proficiency of special education professionals in vocabulary assessment [52].

In conclusion, the combined analysis of satisfaction levels and demographic differences provides a comprehensive understanding of professionals’ perceptions and identifies key areas for improvement. By addressing these challenges through targeted interventions, policy adjustments, and tailored training programs, the overall satisfaction and effectiveness of vocabulary assessments in Saudi Arabia can be significantly improved.

### Limitations

There are some limitations to this study, despite the valuable insights it provides. The use of self-reported data introduces the potential for response bias, underscoring the importance of exercising caution when interpreting the findings. For example, most of the responses were received from particular groups such as professionals with less than 10 years of experience. Moreover, the study’s cross-sectional design imposes limitations on the ability to establish causal relationships.

To gain a more comprehensive understanding of the changing dynamics of satisfaction among special education professionals, future research should consider using longitudinal designs. This approach would allow for the examination of satisfaction over an extended period, enabling researchers to observe how it evolves and develops [25]. By employing this method, a more nuanced understanding of the factors influencing satisfaction in this field can be achieved. Gaining insight into the dynamics of satisfaction levels over time and discerning the factors that influence these changes would offer a more comprehensive understanding.

### Recommendations for future research

In light of the current study, it is recommended that future research endeavors focus on investigating the efficacy of targeted training modules in improving satisfaction levels among professionals. A thorough examination of the lasting effects of training on vocabulary assessment practices and student outcomes would yield a better understanding of the enduring advantages of professional development initiatives. The inclusion of comparative studies conducted in various regions and cultural contexts has the potential to enhance our understanding of the challenges and achievements associated with vocabulary assessment in special education. By examining these diverse perspectives, we can develop a more comprehensive and globally informed viewpoint on this matter.

## Conclusion

This investigation of satisfaction levels with vocabulary assessment among professionals in Saudi Arabia does much to provide an informative overview of the current scenario. The detailed findings strongly call for specific interventions and, even more so, training and professional growth. The differences found among individuals based on the type of major selected highlight the need for targeted strategies to address the distinct issues faced by professionals in various domains of special education. As Saudi Arabia continues its efforts to implement an inclusive education agenda, these findings offer invaluable insights that serve as guidelines for policy and practice improvement, potentially shaping experiences for students with disabilities in ways that better support an ideal educational environment. It is of paramount importance for special education professionals to intensify their efforts in research, training, and collaboration to adequately support this diverse student population. These efforts are essential in equipping professionals with the necessary skills and knowledge required to navigate the complexities of assessing vocabulary. Investing in such activities enhances the quality of support provided to students.

## Supporting information

S1 File Satisfaction Questionnaire of Vocabulary Assessment (SQVA). (PDF)
