# Correction: The impact of vocabulary assessments on quality of life: Insights from professionals on their application with students with disabilities

**DOI:** 10.1371/journal.pone.0330198

**Published:** 2025-08-11

**Authors:** Faisl Alqraini, Khalid Alasim, Abdulaziz Alqahtani

In the Acknowledgment section, the funding acquisition attributed to the author Faisal Alqraini should have been removed.
