# Supplementary material for: The impact of vocabulary assessments on quality of life: Insights from professionals on their application with students with disabilities
Source: PLoS One. 2024 Nov 12;19(11):e0313690. doi: 10.1371/journal.pone.0313690 (PMC11556738; doi:10.1371/journal.pone.0313690)
Supplement: S1 File — (PDF) [file pone.0313690.s001.pdf]

Satisfaction Questionnaire of Vocabulary Assessment (SQVA)

Demographic Characteristics of the Participant

1. **Gender:**

- ❍ Male
- ❍ Female

2. **Sectors:**

- ❍ Private
- ❍ Government
- ❍ Hospital.

3. **What is the highest level of formal education you have completed?**

- ❍ Diploma
- ❍ Completed bachelor’s degree
- ❍ Completed graduate school (MA or PhD)

4. **Major:**

- ❍ Speech and Communication Disorders
- ❍ General Special Education
- ❍ Deaf and Hard of Hearing

5. **Type of disability you work with:**

- ❍ Deaf and hard of hearing
- ❍ Learning disabilities
- ❍ Speech and communication disorder

- ❍ Blind
- ❍ Intellectual disability
- ❍ Autism
- ❍ Behavioral and emotional disorders

6. Years of experience working with disability students:

- ❍ Less than 5 years
- ❍ Between 5-10 years
- ❍ More than 10 years

7. Region:

- ❍ Western region
- ❍ Eastern region
- ❍ Middle region
- ❍ Southern region
- ❍ Northern region

8. Training and professional development in vocabulary assessment:

- ❍ One training
- ❍ More than one training
- ❍ None

Instrument

Survey item	Very Dissatisfied	Dissatisfied	Neither Satisfied nor Dissatisfied	Satisfied	Very Satisfied	Not Applicable
1. The official vocabulary assessments are easy to use.	○	○	○	○	○	○
2. The vocabulary assessments measure the actual level of the student.	○	○	○	○	○	○
3. Clarity of instructions on how to use the vocabulary assessments.	○	○	○	○	○	○
4. The vocabulary assessments measure the	○	○	○	○	○	○

language level of elementary school students.						
5. The vocabulary assessments facilitate parents' recognition of their children's language needs in acquiring vocabulary. professionals.	○	○	○	○	○	○
6. The vocabulary assessments are electronically automated.	○	○	○	○	○	○
7. The vocabulary	○	○	○	○	○	○

assessments contribute to identifying the strengths and weaknesses of the student in language.						
8. The results of the vocabulary assessments are effectively employed in building the appropriate educational and therapeutic program for the student.	○	○	○	○	○	○
9. Currently, the translated and standardized	○	○	○	○	○	○

vocabulary assessments are outdated.						
10. The vocabulary assessments are suitable for the Saudi environment.	○	○	○	○	○	○
11. Overall, satisfaction of vocabulary assessments.	○	○	○	○	○	○

**Comments and Suggestions:**
**Thank you for your participation**
